# Malignant mesothelioma of the tunica vaginalis testis: a malignancy associated with recurrent epididymitis?

**DOI:** 10.1186/1477-7819-10-238

**Published:** 2012-11-09

**Authors:** Ching-Heng Yen, Chun-Te Lee, Chung-Jen Su, Hua-Cheng Lo

**Affiliations:** 1 Division of Urological Surgery, Department of Surgery, Song-Shan Armed Forces General Hospital, No. 131, JianKang Rd., SongShan Dist., Taipei City, 105, Taiwan

**Keywords:** Malignant mesothelioma of the tunica vaginalis testis, Epididymitis, Hydrocele

## Abstract

A 53-year-old Taiwanese male had several episodes of left epididymitis with hydrocele refractory to antibiotic treatment. Partial epididymectomy plus preventive vasectomy were planned, and, incidentally, an ill-defined nodule was found lying on the tunica vaginalis near the epididymal head. The pathological diagnosis was malignant mesothelioma of the tunica vaginalis testis. Radical orchiectomy with wide excision of the hemi-scrotal wall was performed. So far, there is no evidence of recurrence after more than 3 years of follow-up. Malignant tumor should be considered in the case of recurrent epididymitis refractory to empirically effective antibiotic treatment. Although the nature of this tumor is highly fatal, the malignancy can possibly be cured by early and aggressive surgical treatment.

## Background

Malignant mesothelioma of the tunica vaginalis testis is a rare tumor. Since the first case report, fewer than 100 cases have been reported to date [1,2]. Most of these cases presented with nonspecific symptoms, such as a painless scrotal mass associated with hydrocele, even in the advanced stage. The pathogenesis of this malignant neoplasm is unclear and is mostly related to asbestos exposure [3], as well as a long-lasting hydrocele [4]. An early preoperative diagnosis is difficult to obtain, and a few cases have even been suspected to be epididymitis [5]. We hereby report a case of malignant mesothelioma of the tunica vaginalis testis that mimicked epididymitis with hydrocele.

## Case presentation

**Figure 1:**
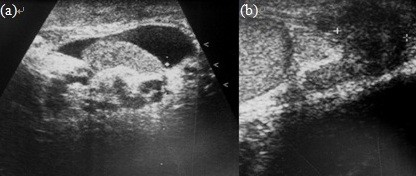
**Scrotal ultrasound image from 1 year ago (A) and prior to surgery (B).** (**A**) mild hydrocele and increased thickness of the left tunica vaginalis; (**B**) a hypoechoic lesion of approximately 0.5 cm in size over the left epididymal head.

About 3 years ago, a 53-year-old man was referred to our hospital with the chief complaint of a repetitive painful nodule over the left scrotum. He had visited a local medical clinic and had been treated with empirical antibiotics with the diagnosis of epididymitis. The patient was sexually active and reported no lower urinary tract symptoms. He denied any history of exposure to asbestos or scrotal trauma. First, scrotal ultrasonography was arranged to rule out an abscess. The ultrasonographic pictures (Figure 1A) showed a left scrotal mild hydrocele and an increased thickness of the scrotal wall as seen in epididymitis. Following modification of antibiotic treatment, the symptoms subsided for about one year.

One year later the patient returned with the problem of several episodes of scrotal pain and recurrent epididymitis. This time, antibiotic therapy had a limited therapeutic effect, and the inflammatory symptoms fluctuated, but never subsided. Thus, we decided to perform partial epididymectomy plus a preventive vasectomy. Prior to surgery, follow-up scrotal ultrasonography demonstrated a hypoechoic lesion of about 0.5 cm in size over the left epididymal head (Figure 1B), and inflammatory granuloma was suspected. During the operation, an ill-defined nodule (measuring 2 × 1 × 1 cm) was found lying on the tunica vaginalis near the epididymal head. The nodule was excised from the tunica vaginalis and a partial epididymectomy plus a preventive vasectomy were performed.

**Figure 2:**
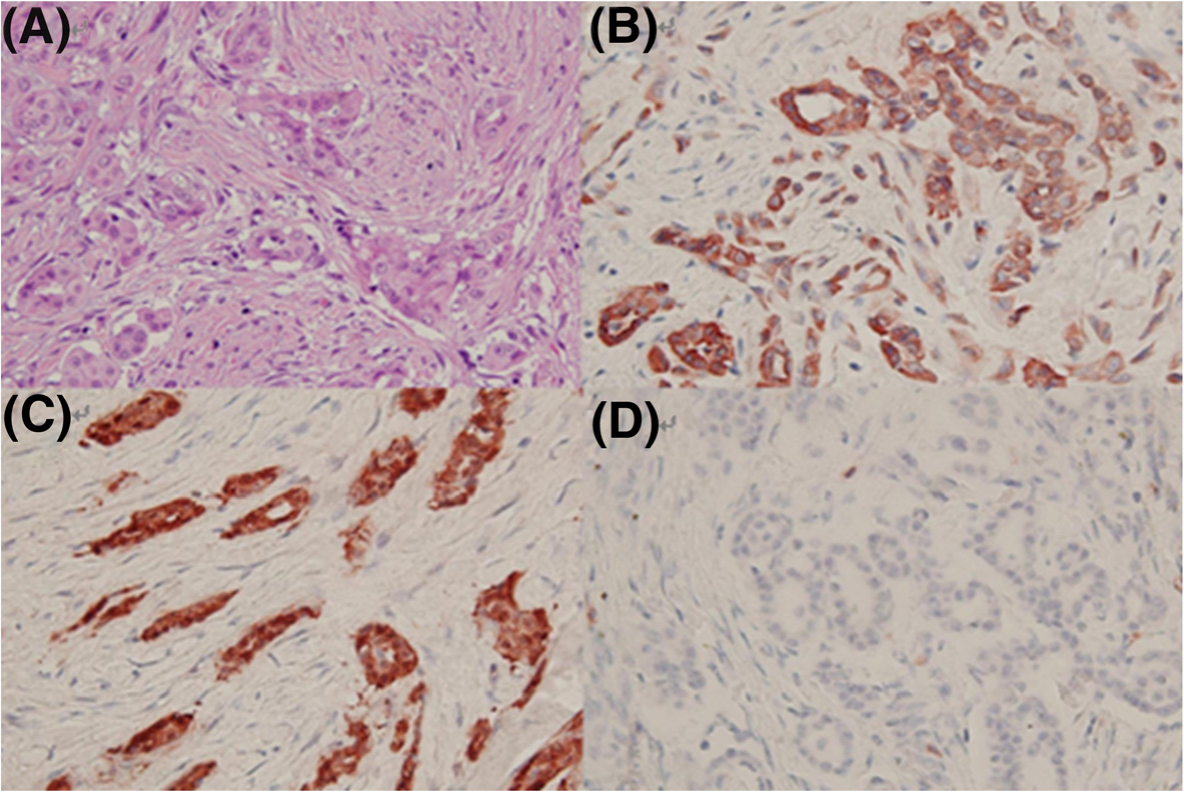
**Histological and immunohistochemical features of malignant mesothelioma of the tunica vaginalis testis.** (**A**) The tumor cells are rounded or cuboidal with nuclear atypia, prominent nucleoli and a diffuse infiltrating pattern (haematoxylin and eosin stain × 100); (**B**) positive for cytokeratin stain; (**C**) positive for calretenin stain; (**D**) negative for CEA stain.

The pathological report showed unusual neoplastic change of the tunica vaginalis. Under microscopic examination, the nodule showed a picture of malignant mesothelioma, well-differentiated, accompanied by tubulopapillary morphological features. These tumor cells were rounded or a cuboidal pattern with nuclear atypia, prominent nucleoli and a diffuse infiltrating pattern (Figure 2A). By immunohistochemical staining, the cells were found to be consistently positive for cytokeratin stain and calretenin stain but negative for CEA stain (Figure 2B-D), and the diagnosis of malignant mesothelioma of the tunica vaginalis testis was confirmed.

Once the pathologic diagnosis was established, complete workup of the tumor staging was arranged. By computerized tomography, no evidence of local invasion or metastasis in the adjacent lymph nodes was demonstrated. Subsequently, radical orchiectomy with wide excision of the hemi-scrotal wall was performed. The patient was discharged and so far (after over 3 years of follow-up) there is no evidence of local or distant recurrence.

## Discussion

Malignant mesothelioma of the tunica vaginalis testis is an uncommon disease and is usually described as originating from the pleura, pericardium, and peritoneum. Malignant mesothelioma of the tunica vaginalis testis is extremely rare. Since the first case was described in 1957 [6], about 100 cases have been reported to date. Although this tumor is most often seen in patients between the ages of 55 and 75 years, 10% of patients are younger than 25 years [5]. The pathogenesis of malignant mesothelioma has a strong relationship with occupational exposure to asbestos and long-lasting hydrocele. However, several cases including ours have been reported, in which there is no history of exposure to asbestos. In a review of non-asbestos-related malignant mesothelioma, Peterson and associates [7] found that chronic inflammatory processes have also been suggested to be possible causes of malignant mesothelioma. Previous histories of lung diseases, such as recurrent acute lung infections, active pulmonary tuberculosis, recurrent pleural effusion of unknown origin, and recurrent spontaneous pneumothorax, have been noted in patients who are victims of pleural malignant mesothelioma and have never been exposed to asbestos. In addition, in the peritoneum, malignant mesothelioma also occurs in patients with repeated pneumoperitoneum resulting from pulmonary tuberculosis, those with a long history of severe persistent and recurrent diverticulitis, and those with a long history of familial Mediterranean fever with recurrent peritonitis.

The main clinical symptom of these patients with malignant mesothelioma of the tunica vaginalis testis is a painless scrotal mass with hydrocele. Diagnosis is mostly made intraoperatively or postoperatively owing to the nonspecific symptoms and the absence of a tumor marker before surgery. By reviewing evidence obtained by searching PubMed for articles on malignant mesothelioma of the tunica vaginalis testis between 1998 and August 2012 [1-5,8-33], and including our case, we studied a total of 101 cases to analyze the possible clinical manifestations or preoperative diagnoses of this malignancy (Table 1). The common preoperative impressions or diagnoses were hydrocele (49.5%), suspicious testicular tumor (36.6%), inguinal hernia (5.9%), epididymitis (3%), spermatocele (2%), testicular torsion (2%), and post-traumatic testicular lesion (1%). Epididymitis is rarely presented in malignant mesothelioma of the tunica vaginalis testis, and only three cases have been reported.

**Table 1:** Common preoperative diagnoses of mesothelioma of the tunica vaginalis testis

**Preoperative diagnosis**	**Case number**	**Percentage (%)**	**Reference**
Hydrocele	50	49.5	[1-5,8,10,14-16,20,21,23,32,33]
Testicular tumor	37	36.6	[3,5,9,13,17,18,21-26,28-31]
Inguinal hernia	6	5.9	[5,11,23,27]
Epididymitis	3	3	[5,12], and our study
Spermatocele	2	2	[5]
Testicular torsion	2	2	[19,27]
Post-traumatic testicular lesion	1	1	[5]
Total number	101	100	

In general, there are several causes of epididymal inflammation: infection, trauma, autoimmune disease, vasculitis, and idiopathic. The pathophysiology of epididymitis remains unclear, although it is postulated to occur secondary to retrograde flow of infected urine into the ejaculatory duct. Epididymitis is characterized by inflammation of the epididymis presenting as pain and swelling, generally occurring on one side and developing over several days. Multiple objective findings of epididymitis have been identified and, in variable degrees may include positive urine cultures, fever, erythema of the scrotal skin, leukocytosis, urethritis, hydrocele, and involvement of the adjacent testis [34]. The inflammation of the epididymis frequently involves the surrounding tunica vaginalis and induces reactive hydrocele and serosal injury. Persistent serosal injury, as in epididymitis, hydrocele, hematocele, and inguinal hernia sacs, may cause reactive hyperplasia of the mesothelial lining and submesothelial fibrosis. The combination of the two responses may easily result in histological characteristics mimicking malignant mesothelioma, papillae and small tubules, solid nests and cords extending into the underlying reactive connective tissue, simulating invasion [35]. Following long-term or repetitive inflammatory stimuli, the reactive mesothelial hyperplasia might possibly be further transformed into malignant mesothelioma. Therefore, like pleural and peritoneal malignant mesothelioma, chronic inflammation/infection might provide a hypothetical mechanism of tumorigenesis linking recurrent epididymitis with malignant mesothelioma of the tunica vaginalis testis.

Because there is no precise diagnostic modality available, several methods have been applied for early detection prior to surgery. The most popular diagnostic tool is scrotal ultrasound. A series of sonographic features in scrotal mesothelioma were reviewed for preoperative diagnosis. In addition to hydrocele being commonly presented, scrotal mesothelioma could be associated with an extratesticular mass with atypical sonographic features [36]. Color Doppler ultrasound might have the potential to be used to unveil the vascular characteristics of malignant mesothelioma [32,37]. However, either hypervascularity or hypovascularity in mass lesions has been described in articles, and thus color Doppler ultrasound could not be used as a definitive diagnostic tool. Preoperative diagnosis using aspiration cytology of the hydrocele fluid has been described in cases of rapid-growing hydrocele in the elderly [38]. Considering the sensitivity of cytology and the potential risk of metastasis, the routine use of fine needle aspiration for preoperative diagnosis is still under debate. Therefore, there seems to be no definite diagnostic modality established prior to surgery, and so far definite diagnosis usually warrants surgical exploration as well as pathological examination with special immunohistochemical stains.

Treatment for malignant mesothelioma of the tunica vaginalis testis is still not established. Some authors have adopted the treatment of pleural mesothelioma [39] for patients with paratesticular malignant mesothelioma, but this is of limited therapeutic efficacy. Surgery should be the first-line therapy in cases of early disease. Radical inguinal orchidectomy appears to be the optimum treatment. Local resection of the hydrocele wall is associated with a local recurrence rate of 36%, and hemiscrotectomy is often required for local control, whereas local recurrence after orchidectomy is reported in 10.5 to 11.5% of patients [5]. Adjuvant therapy with systemic chemotherapy and radiotherapy might provide a better overall survival rate in advanced disease [40], but this has not been fully assessed yet. Malignant mesothelioma of the tunica vaginalis testis is an aggressive tumor with high recurrence and mortality rates; about 52% of patients develop local recurrence or metastasis and 40% of patients die from their disease, with a median survival of 24 months [5,41]. Age (< 60 years) and organ-confined disease at diagnosis are significant factors correlated with survival [5]. As our patient has remained well and free of recurrence or metastasis for more than 3 years, we believe that early aggressive surgical excision (radical orchiectomy with wide excision of the hemi-scrotum) is effective and necessary for successful treatment.

## Conclusion

In this case report, we have demonstrated that malignant mesothelioma of the tunica vaginalis testis might mimic epididymitis. We also proposed a hypothetical correlation between epididymitis and paratesticular malignant mesothelioma. Malignancy should be considered in the differential diagnoses, especially in cases of recurrent epididymitis refractory to empirically effective antibiotic treatment. Definitive diagnosis warrants surgical exploration. Once a pathological diagnosis is confirmed, radical orchiectomy with hemiscrotectomy should be performed as soon as possible. Although the nature of this tumor is highly fatal, the malignant disease could be potentially cured by early and aggressive surgical treatment.

## Consent

Written informed consent was obtained from the patient for publication of this case report and any accompanying images. A copy of the written consent is available for review by the Editor-in-Chief of this journal.

## Competing interests

The authors declare that they have no competing interests.

## Authors’ contributions

CHY and HCL were responsible for delivering patient care. CTL and CJS provided opinions in diagnosis and treatment. CHY contributed towards to drafting the manuscript while HCL provided overall supervision and edited the final version of the manuscript. All authors read and approved the final manuscript.

## Authors’ information

Division of Urological Surgery, Department of Surgery, Song-Shan Armed Forces General Hospital, Taipei, Taiwan.
